# Tsunami-generated magnetic fields have primary and secondary arrivals like seismic waves

**DOI:** 10.1038/s41598-021-81820-5

**Published:** 2021-01-27

**Authors:** Takuto Minami, Neesha R. Schnepf, Hiroaki Toh

**Affiliations:** 1 Graduate School of Science, Kobe University, Nada-ku, Kobe, 6578501 Japan; 2 Cooperative Institute for Research in Environmental Sciences (CIRES), University of Colorado, Boulder, CO 80309-0216 USA; 3 Department of Geological Sciences, University of Colorado, Boulder, CO 80309-0216 USA; 4 Graduate School of Science, Kyoto University, Sakyo-ku, Kyoto, 6068502 Japan

**Keywords:** Natural hazards, Geomagnetism, Physical oceanography

## Abstract

A seafloor geomagnetic observatory in the northwest Pacific has provided very long vector geomagnetic time-series. It was found that the time-series include significant magnetic signals generated by a few giant tsunami events including the 2011 Tohoku Tsunami. Here we report that the tsunami-generated magnetic fields consist of the weak but first arriving field, and the strong but second arriving field—similar to the P- and S-waves in seismology. The latter field is a result of coupling between horizontal particle motions of the conductive seawater and the vertical component of the background geomagnetic main field, which have been studied well so far. On the other hand, the former field stems from coupling between vertical particle motions and the horizontal component of the geomagnetic main field parallel to tsunami propagation direction. The former field has been paid less attention because horizontal particle motions are dominant in the Earth’s oceans. It, however, was shown that not only the latter but also the former field is significant especially around the magnetic equator where the vertical component of the background magnetic field vanishes. This implies that global tsunami early warning using tsunami-generated magnetic fields is possible even in the absence of the background vertical geomagnetic component.

## Introduction

**Figure 1:**
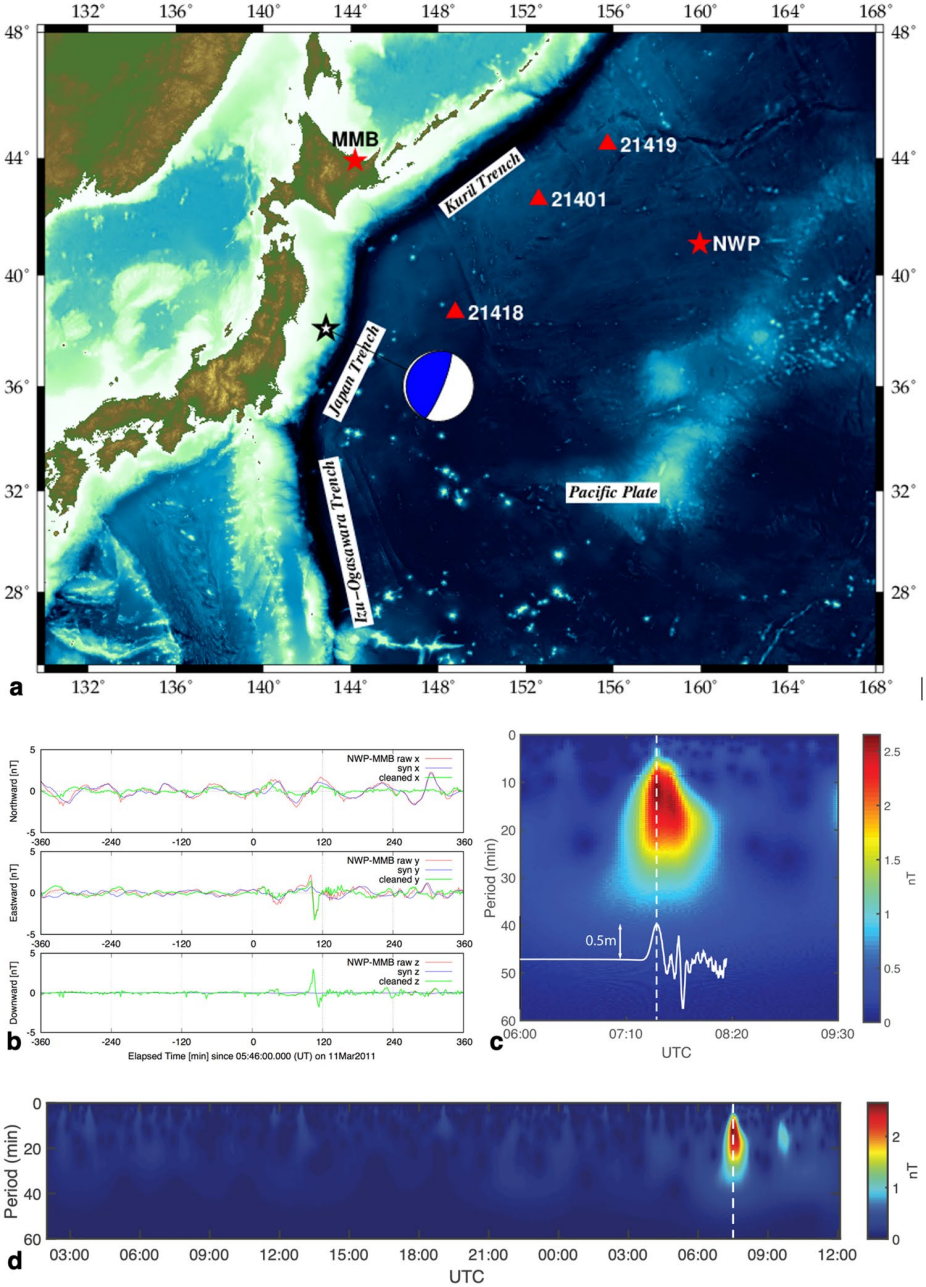
Site map, observed time-series and cross-wavelet analysis results. (**a**) The focal mechanism (the beach ball*) of the 2011 off the Pacific coast of Tohoku Earthquake and its epicenter. Stars indicate the locations of our seafloor EM station (NWP) and the reference geomagnetic observatory on land (MMB). Triangles show the closest three DART** buoys. This figure was newly created by one of the authors (T.M.) using a combination of public domain software and data, viz., Generic Mapping Tools (GMT)^24^ v.6.1.1 (https://www.generic-mapping-tools.org/) and the global 1 min digital topography v.19.1 (https://topex.ucsd.edu/WWW_html/mar_topo.html). (**b**) High-passed raw vector geomagnetic data (red), synthetic variations of external origin (blue), and the difference (green = red–blue) at NWP. The synthetic variations were calculated using the transfer function that casts the external geomagnetic field at MMB to that at NWP. The raw geomagnetic data were high pass filtered with a cut-off period of 2 h. (**c**) The 3.5-h periodogram by our cross-wavelet analysis method^17^ for the difference data of *b*_z_ (the bottom green curve in **b**). This analysis used a maximum cut-off period of 30 min. The vertical dashed line indicates the estimated time of arrival of the tsunami at NWP (07:30 UTC). The white line shows a result of kinetic simulation for the 2011 Tohoku tsunami. (**d**) Same as (**c**) but for a longer duration (21 h) to show the non-tsunami-related variations as well. *https://earthquake.usgs.gov/earthquakes/eventpage/official20110311054624120_30/moment-tensor **https://www.ndbc.noaa.gov/dart.shtml.

A devastating tsunamigenic earthquake of Mw9.1 occurred on the landward slope of the Japan Trench on March 11, 2011 (Table 1), which resulted in enormous damage to Japanese society not only by its very strong seismic motions but also its gigantic tsunami. Seismic and tsunami data of this large earthquake have been studied intensively to yield its focal and tsunami source mechanisms^1^. Its tsunami was also detecteded^2,3^ by the seafloor geomagnetic observatory^4,5^ operating on the northwest Pacific Basin even at a large epicentral distance of more than 1500 km (Fig. 1). It was found that the seafloor observatory detected significant magnetic signals generated by a few giant tsunami events^6,7^ including that of the 2011 Tohoku Earthquake^2^. Those detections were enabled through the so-called motional induction effect^8^, which was first studied by Faraday^9^. Since then, study of this effect had been mainly focused on non-transient oceanic motions such as tides^10–13^ and the western boundary currents^14–16^. However, the time scale of tsunamis is much shorter than that of the long-period currents and thus temporal variations of the tsunami-generated magnetic fields should be considered explicitly in solving the induction equation for magnetic fields in either the frequency^6^ or time ^2,3^ domain.

**Table 1:** Earthquake and site descriptions.

Latitude (^o^N)	Longitude (^o^E)	Depth (km)	Origin time	Moment magnitude (Mw)
			Epicentral distances (km)	Site names
38.297	142.373	29.0	March 11, 2011 05:46:24 UTC	9.1*
41.102	159.963	5.58	1535.8	NWP
43.910	144.189	− 0.042	643.1	MMB

*Details of the tsunamigenic earthquake can be obtained from United States Geological Survey (USGS) at https://earthquake.usgs.gov/earthquakes/eventpage/official20110311054624120_30/executive.

## Results

Figure 1b clearly shows that the 2011 off the Pacific coast of Tohoku Earthquake emitted tsunamis that can be identified by magnetic variations as large as 3nT even at a very large epicentral distance (1536 km; Table 1). The time variations are evident mainly in the eastward and downward magnetic components, because the tsunamis propagated towards east from the epicentre to the seafloor site (NWP; Fig. 1a and Fig. S1a). At NWP, the magnetometer sensed the eastward and downward components since the tsunami-generated electric currents were concentrated along the tsunami wave front that oriented in the north–south direction around NWP (Fig. S1b). Even though geomagnetic variations on the seafloor are less subject to external fields, the observed raw time series (the red curves in Fig. 1b) were corrected by a transfer function method^4^ between NWP and a remote reference site (MMB) using very long time-series of non-tsunami periods to yield cleaned time-series (the green curves). It has been pointed out that the wavelet analysis is a powerful tool to detect geomagnetic disturbances^17,18^. The cleaned time-series, therefore, were further analysed by a cross-wavelet analysis method (Fig. 1c), which successfully identified the co-tsunami magnetic variation even in the presence of a moderate external disturbance manifested in *K*_p_ indices^19^ on March 11, 2011. It is evident in Fig. 1d that there exist no significant signals in the cross-wavelet result before the tsunami arrival.

The presence of a significant tsunami-generated magnetic signal in the cleaned time-series is indisputable as shown in Fig. 1c. However, the phase relation between the tsunami wave height and the tsunami-generated magnetic field is not very clear in this plot. We, therefore, used an analytical solution of the tsunami-generated electromagnetic (EM) fields in the frequency domain to clarify whether the magnetic field has an identifiable phase lag or lead with respect to tsunami wave height. Detailed derivation of the analytical solution utilized here is described in the ‘Methods Section’.

Analytical solutions of tsunami-generated EM fields in the frequency domain have been obtained by several works^20–22^. However, the majority of the preceding works neglected the source electromotive force arising from coupling of the horizontal geomagnetic component (*F*_y_) with the vertical flow velocity (*v*_z_) of the conductive seawater. Instead, they focused on the coupling of the vertical geomagnetic component (*F*_z_) with the horizontal flow velocity (*v*_y_), which may have been a natural choice, to first-order approximation, because *v*_y_ is several times larger than *v*_z_ in the case of tsunamis. Our point here, however, is that the *v*_z_*F*_y_ coupling cannot be neglected in the sense that not only does it create a large phase lead of the tsunami-generated EM fields with respect to the kinetic phase of tsunamis but it also does not vanish to zero amplitude—even in the equatorial regions where *F*_z_ tends to become very small.

**Figure 2:**
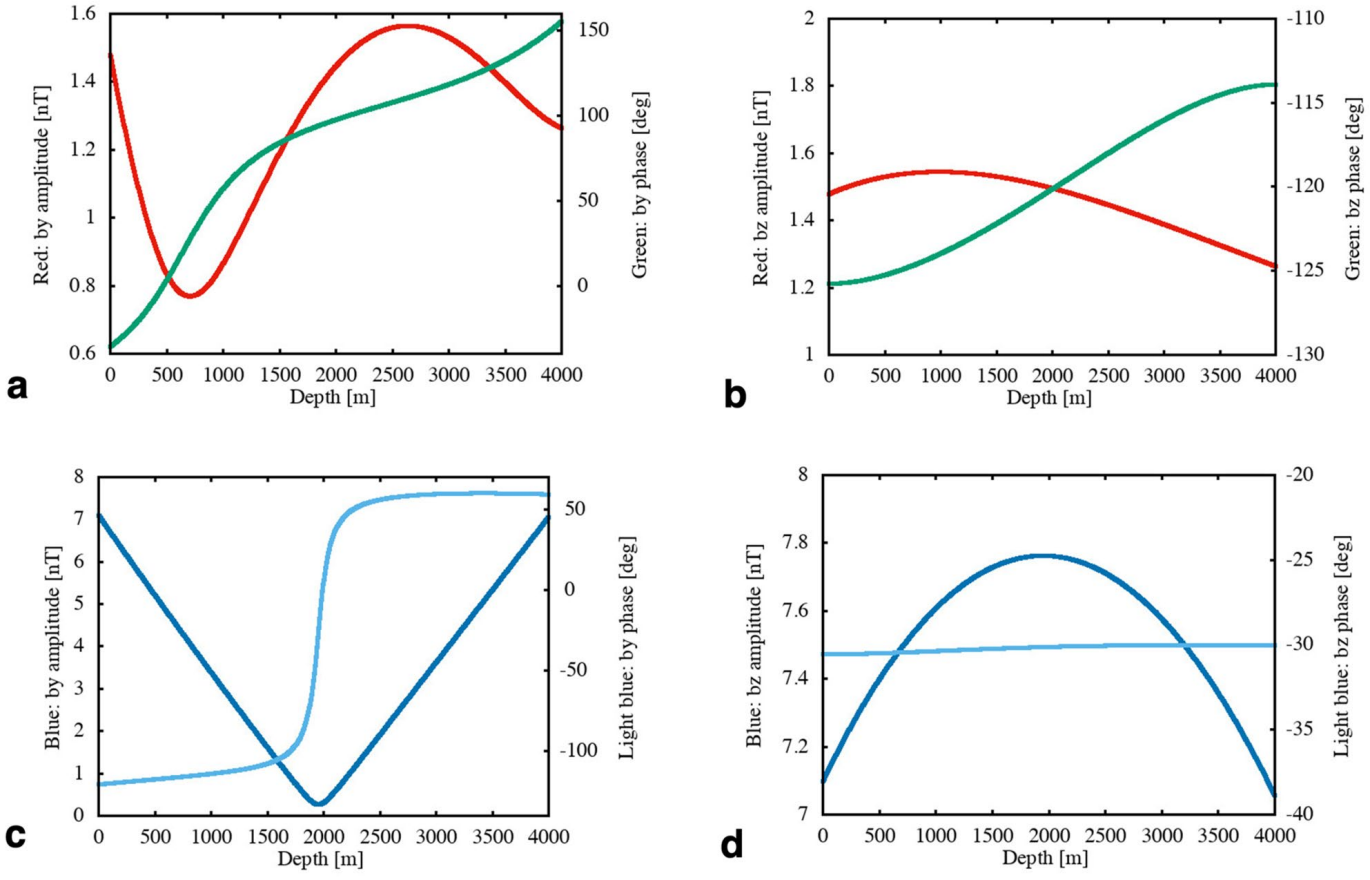
Analytical solutions arising from either *F*_y_ or *F*_z_ of the ambient field. **a,** Amplitude (red) and phase (green) curves of the horizontal magnetic component (*b*_y_) generated by the *v*_z_*F*_y_ coupling within a flat ocean of 4000 m depth over a uniform half space of 0.01S/m conductivity. Phases are given as ‘lag’ w.r.t. the maximum tsunami wave height. (**b**) Same as (**a**) but for the downward magnetic component (*b*_z_). (**c**) Amplitude (blue) and phase (light blue) curves of the horizontal magnetic component (*b*_y_) generated by the *v*_y_*F*_z_ coupling. This component retains opposite signs and the maxima of amplitudes on both sides of the ocean. The presence of conductive substrata results in a slight deviation from the perfect symmetry with respect to the half ocean depth (2000 m). (**d**) Same as (**c**) but for the downward magnetic component (*b*_z_). Note that the sign of ${b}_{z}$ by the ${v}_{z}{F}_{y}$ coupling is flipped to make it correspond to the peak of ${v}_{z}$ that has a π/2 phase lead w.r.t. tsunami wave height. The wave height, *A*, is 1 m and the frequency is 3 mHz. We used 4S/m for the electrical conductivity of the seawater and assumed the strength of the ambient geomagnetic field to be *F*_y_ = *F*_z_ = 35,000nT.

We plotted the contribution of the *v*_z_*F*_y_ coupling in the upper two panels of Fig. 2, while the *v*_y_*F*_z_ coupling is shown in the lower panels. The magnitudes of the ambient geomagnetic components were set identical for both couplings for easier comparison. The left two panels of Fig. 2 show the *b*_y_ component, while the right panels show *b*_z_. Comparing the amplitudes of the tsunami-generated magnetic components, the *v*_z_*F*_y_ coupling gives ~ 1.5nT at most, whereas the *v*_y_*F*_z_ coupling can create 7 ~ 8nT—4–5 times more than the *v*_z_*F*_y_ coupling. All of the tsunami-generated magnetic components are subject to significant changes in amplitude through the ocean layer. Especially for the *v*_z_*F*_y_ coupling, the depth dependence of the tsunami-generated magnetic components in a uniformly conducting ocean is governed by a combination of the different reflection of the EM fields in the ocean at the seafloor and at the sea surface, and the almost linear decrease of *v*_z_ with depth from a finite value at the sea surface to nil at the seafloor. It is noteworthy that the amplitude ratio, *b*_*z*_ (or *b*_*y*_) by the *v*_*y*_*F*_*z*_ coupling to those by the *v*_*z*_*F*_*y*_ coupling, is nearly equal to that of the flow velocity (*v*_y_/*v*_z_ ~ 5).

The phase lag of the tsunami-generated magnetic components with respect to the tsunami wave height also has depth dependence. According to Fig. 2a–d, every component has larger lags at depth compared with those near the sea surface. Note that the phase difference is given as ‘phase lag’ in Fig. 2, so the negative values actually correspond to ‘phase lead’. The *b*_z_ component arising from the *v*_y_*F*_z_ coupling (Fig. 2d) possesses the largest amplitude (~ 7.8 nT in the middle of the ocean layer). However, its phase lead is only 30 degrees for the 3 mHz frequency used in the calculation. This means that the largest tsunami-generated magnetic signal arrives just before the actual tsunami arrival. The phase lead is equivalent to only a one-twelfth of the tsunami period, which is equal to slightly less than 28 s. The same argument is applicable to the *b*_y_ component arising from the *v*_z_*F*_y_ coupling (Fig. 2a), which possesses large phase lags in most of the ocean layer but turns to ‘lead’ of less than 40 degrees near the sea surface.

The largest phase lead is realized by the *b*_z_ component arising from the *v*_z_*F*_y_ coupling (Fig. 2b). It reaches a lead as large as 125 degrees—equivalent to more than one-third of the tsunami period. Namely, we can observe a small but significant (more than 1 nT) tsunami-generated magnetic signal 116 s (~ 2 min) prior to the actual tsunami arrival in this case. Another significant phase lead is achieved by the *b*_y_ component arising from the *v*_y_*F*_z_ coupling (Fig. 2c), which possesses a large amplitude (~ 7nT) but the large phase lead (~ 120 degrees) can only be observed on the sea surface and not on the seafloor. The reason why the *v*_z_*F*_y_ coupling leads the *v*_y_*F*_z_ coupling by approximately 90 degrees in phase lies in the fact that *v*_z_ has maximum amplitude at the wave’s nodes (see Fig. S2) while *v*_y_ does at the peaks.

**Figure 3:**
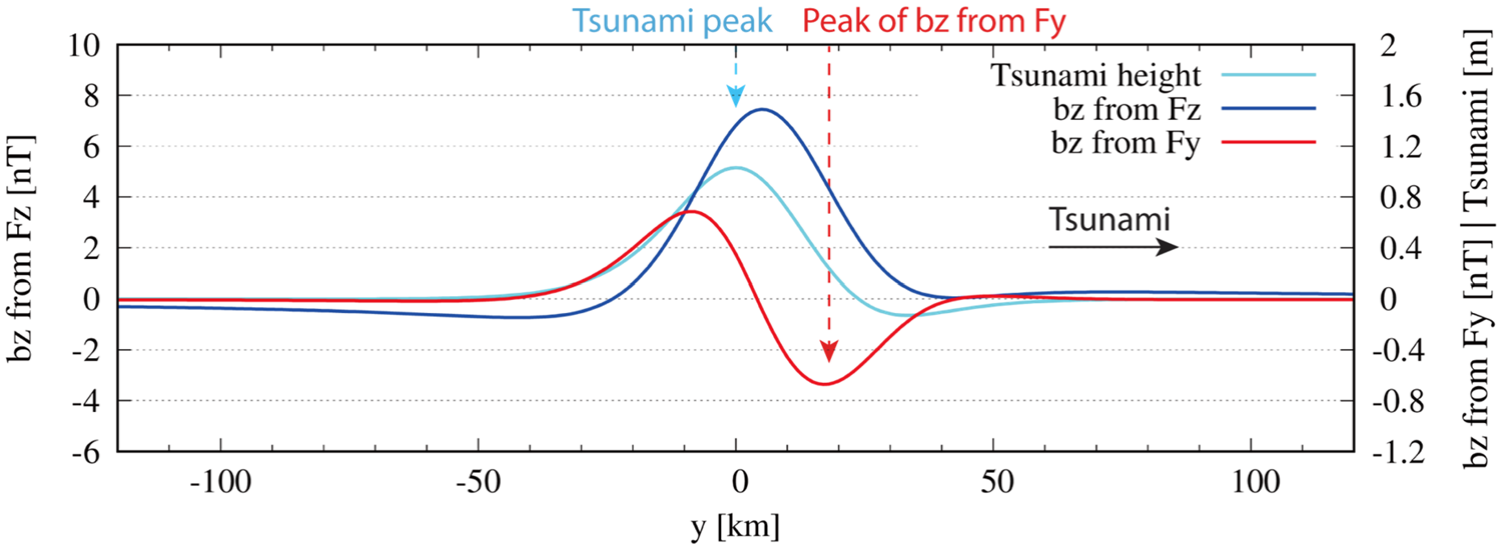
Predicted phase lead of ${b}_{z}$ by the ${{v}_{z}F}_{y}$ coupling of the solitary wave. Predicted vertical components of the magnetic variation at the seafloor, caused by coupling of ${v}_{y}{F}_{z}$ (${b}_{z}$ from ${F}_{z}$) and ${v}_{z}{F}_{y}$ (${b}_{z}$ from ${F}_{y}$). Refer to the left ordinate for ${b}_{z}$ from ${F}_{z}$ and right for both ${b}_{z}$ from ${F}_{y}$ and the tsunami height. ${F}_{y}={F}_{z}=35000\mathrm{nT}$ are adopted in the calculation. Ocean depth is set to 4000 m. See the “Methods” section for the derivation of the tsunami wave form and how the magnetic variations are calculated. The negative peak of “${b}_{z}$ from ${F}_{y}$” clearly precedes the peak of solitary tsunami and that of “${b}_{z}$ from ${F}_{z}$”.

Actual phase lead of the magnetic variation at the time of tsunami first arrival should be investigated more carefully since the phase relationship mentioned above is based on the continuous sinusoidal tsunami and the analytical solution is given in frequency domain. We, therefore, modelled magnetic variation due to a synthetic (but realistic) tsunami first arrival, which consists of a large main tsunami peak of 1 m and a preceding small negative peak of 0.1 m due to elasticity of the Earth^23^. See “Method” section for details of the solution in time domain. Figure 3 shows the result of the modelling, where ${b}_{z}$ due to the ${v}_{z}{F}_{y}$ and ${v}_{y}{F}_{z}$ couplings are compared with the adopted tsunami waveform. The characteristic frequency of the tsunami first arrival is ~ 3 mHz and the tsunami height of the main peak is ~ 1 m. Results from the modelling, therefore, are comparable to those shown in Fig. 2.

Figure 3 illustrates that ${b}_{z}$ from ${{v}_{z}F}_{y}$ has a definite advantage in its phase lead, though the amplitude and the phase lead are smaller than expected by the analytical solution shown in Fig. 2b. As for ${b}_{z}$ from ${v}_{y}{F}_{z}$, the amplitude reaches 7nT and the phase lead amounts to 6 km distance with respect to the tsunami main peak, namely about 30 s ahead in time. As for ${b}_{z}$ from ${v}_{z}{F}_{y}$, the amplitude is ~ 0.6nT and the negative peak precedes the tsunami main peak by 19 km, namely 96 s (1.6 min) in time. The amplitude is about a half and the phase lead is almost comparable to the results from Fig. 2b. Although the phase lead of ${b}_{z}$ from ${v}_{z}{F}_{y}$ is slightly smaller than that expected by the frequency domain solution, the negative peak is still earlier than the main peak of ${b}_{z}$ from ${v}_{y}{F}_{z}$ more than one and half minutes.

## Discussion

It seems promising to observe the *b*_y_ component arising from the *v*_y_*F*_z_ coupling on the sea surface for the purpose of tsunami early warning. Oceanic islands in the mid- to high-latitudes can be regarded as the ideal locations to observe the large phase lead, provided that the site effect in the vicinity of those islands can be corrected properly. However, the *b*_y_ component arising from the *v*_y_*F*_z_ coupling is inevitably associated with the following two drawbacks: (1) No signal can be detected in the equatorial regions since it consists solely of the *F*_z_ contribution. (2) External geomagnetic disturbances are much more abundant in the horizontal geomagnetic components than in the vertical component, which can mask the tsunami-generated signals easily.

On the contrary, the *b*_z_ component arising from the *v*_z_*F*_y_ coupling is strongest (see Fig. S3) on the magnetic equator in terms of the source electromotive force, and it has the largest phase lead among the four components (see Figs. 2a–d). Furthermore, external geomagnetic disturbances contain less of the vertical component, especially in mid-latitudes. Another source of magnetic noise in the vertical component is the electrical structure-dependent horizontal shear of the electric currents flowing both in the ocean and beneath the seafloor that are induced by external disturbances. However, as depicted in Fig. 1b, the effect of EM induction within both the ocean and the solid Earth can be reduced considerably using magnetic data without tsunami events.

Our time domain solution shown in Fig. 3 has revealed that the phase lead of ${b}_{z}$ by ${v}_{z}{F}_{y}$ is slightly smaller than that in frequency domain but still there. The expected phase lead of ~ 2 min in the analytical solution is reduced to 1.6 min for the tsunami first arrival. We speculate that this comes from the difference between solitary waves and continuous sinusoidal waves; the lack of a large negative peak (~ 1 m) preceding the tsunami main peak results in the phase delay and decreases the amplitude of ${v}_{z}$ and thereby those of ${b}_{z}$ by ${v}_{z}{F}_{y}$. Our time-domain modelling also demonstrated that the ${v}_{z}{F}_{y}$ coupling generates ${b}_{z}$ with a detectable amplitude (~ 0.6 nT on the seafloor and ~ 0.8 nT on the sea surface) and with a larger phase lead than that of ${b}_{z}$ by ${v}_{y}{F}_{z}$. In the vicinity of the geomagnetic equator, this effect plays a dominant role in the tsunami-generated magnetic variation because ${F}_{z}$ diminishes virtually there.

Considering the protection against external disturbances argued above, the *b*_z_ components are better choices than the *b*_y_ components. If we select to observe the tsunami-generated *b*_z_ component either on the sea surface or on the seafloor, we first detect a small magnetic signal stemming from the *v*_z_*F*_y_ coupling earlier than the actual tsunami arrival. The small but significant signal will be followed by a much larger signal arising from the *v*_y_*F*_z_ coupling that will be observed just before the tsunami arrival. Those primary and secondary arrivals of the tsunami-generated *b*_z_ component are quite similar to the well-known P- and S-arrivals of seismic waves and can be observed both on the sea surface and on the seafloor.

## Conclusions

Generation of EM fields by conductive geofluid moving through the ambient geomagnetic field has been studied, since the EM induction phenomenon itself was first discovered^9^. Many types of ocean flows, such as tides or the western boundary currents, in addition to tsunamis^3^ have been proved to have motionally induced EM fields. However, most of previous works were focused on the vertical component of the ambient geomagnetic field, neglecting the horizontal geomagnetic component. Here we show that the coupling of the horizontal geomagnetic component with the vertical particle motion of the conductive seawater can generate observable vertical magnetic signals with significant phase lead to the tsunami wave height. Furthermore, we find that the vertical magnetic signal is followed by another vertical magnetic signal of larger amplitude just before the tsunami arrival. This pair of vertical magnetic signals generated by tsunamis is analogous to the pair of seismic P- and S-waves generated by earthquakes. Because those vertical magnetic signals have better protection against geomagnetic disturbances of external origin and the fast-arriving signal is ubiquitous over the globe (i.e., it never vanishes even on the dip equator), the pair of magnetic signals can be applied to global tsunami early warning systems for disaster mitigation. Even though a few minutes seem too short, they are long enough to give alerts for people at the coast to evacuate.

## Methods

### Analytical solution of tsunami-generated EM fields for linear dispersive waves

Analytical solutions of tsunami-generated EM fields in the frequency domain can be derived if analytical forms of the tsunami’s velocity fields are known. Here we adopt two-dimensional linear dispersive waves with wave fronts parallel to the *x*-direction and propagating toward the *y*-direction over a flat ocean of a constant depth of *h* with a downward positive *z*-axis. The origin of *z*-axis is set at the averaged sea surface. We further assume that the seawater is incompressible and the flow is irrotational, which means that there exists a velocity potential, *Ф*, for this flow. It follows that *Ф* is a harmonic function that obeys the following Laplace equation:

1 $$\Delta \Phi = \, 0.$$

It can be readily shown that *Ф* has an analytical form of

2 $$\Phi =-iA\frac{\omega }{k}\frac{\mathrm{cosh}\left(k\left(z-h\right)\right)}{\mathrm{sinh}\left(kh\right)}{e}^{i\left(ky-\omega t\right)},$$

provided that the time- and *y*-dependence of *Ф* is ${e}^{i\left(ky-\omega t\right)}$, and that the energy conservation and linear boundary conditions are imposed on both the sea surface and seafloor. The wave number and angular frequency of the linear dispersive wave in concern are denoted by *k* and *ω*, respectively. *A* is the height of the linear dispersive wave. The dispersion relation of the linear dispersive wave is given by.

3 $$\frac{{\omega^{2} }}{{k^{2} }} = \frac{g}{k}\tanh (kh),$$

where *g* is the gravitational acceleration on the Earth’s surface.

The governing equation of the tsunami-generated magnetic field, ***b***, in a medium of constant electrical conductivity, *σ*, can be written as

4 $$\left({\nabla }^{2}-i\omega \sigma {\mu }_{0}\right){\varvec{b}}=-\sigma {\mu }_{0}\nabla \times \left({\varvec{v}}\times {\varvec{F}}\right),$$

where *µ*_0_ is the magnetic permeability of the medium, while ***v*** and ***F*** are the particle velocity of the linear dispersive wave and the ambient magnetic field (|***b***|< <|***F***|), respectively. We consider a one-dimensional Earth model for the electrical conductivity that varies only in the *z*- direction. In the flat ocean, Eq. (4) can be rewritten as:

5 $$\left\{ {\frac{{\partial^{2} }}{{\partial z^{2} }} - \left( {k^{2} - i\omega \sigma_{s} \mu_{0} } \right)} \right\}\left( {\begin{array}{*{20}c} {b_{y} } \\ {b_{z} } \\ \end{array} } \right) = \sigma_{s} \mu_{0} \left( {\begin{array}{*{20}c} { - \frac{\partial }{\partial z}(v_{y} F_{z} - v_{z} F_{y} )} \\ {\frac{\partial }{\partial y}(v_{y} F_{z} - v_{z} F_{y} )} \\ \end{array} } \right).$$

Here, *σ*_s_ is the electrical conductivity of seawater, and *v*_y_ and *v*_z_ can be derived by differentiating *Ф* in Eq. (2) for each spatial direction. In the air and beneath the seafloor, however, the magnetic field obeys either the Laplace or diffusion equation, since the electrical conductivity is nil in the air and the conductors are not moving beneath the seafloor.

The analytical solution in the ocean can be derived by solving Eq. (5) and matching both tangential (*b*_y_) and normal (*b*_z_) components on the sea surface and the seafloor. The solution can be given by the following formulae:

6 $$\begin{aligned} e_{x} + (\vec{v} \times \vec{F})_{x} = - \sqrt {\frac{g}{k}\mathrm{tanh}(kh)} \frac{{kAe^{i(ky - \omega t)} }}{S \cdot \mathrm{sinh}(kh)}\left\{ {\zeta_{x} (z) \cdot F_{z} + i\psi_{x} (z) \cdot F_{y} } \right\}, \hfill \\ \zeta_{x} (z) = - \mathrm{sinh}(\alpha_{S} z) - \frac{{\alpha_{S} }}{k}\mathrm{cosh}(\alpha_{S} z) - e^{kh} \left\{ {P \cdot \mathrm{cosh}(\alpha_{S} (z - h)) - \mathrm{sinh}(\alpha_{S} (z - h))} \right\} = \zeta_{z} (z) - S \cdot \mathrm{cosh}(k(z - h)), \hfill \\ \psi_{x} (z) = P \cdot \left\{ {\mathrm{cosh}(\alpha_{S} z) + \frac{k}{{\alpha_{S} }}\mathrm{sinh}(\alpha_{S} z)} \right\} - e^{kh} \left\{ {P \cdot \mathrm{cosh}(\alpha_{S} (z - h)) - \mathrm{sinh}(\alpha_{S} (z - h))} \right\} = \psi_{z} (z) - S \cdot \mathrm{sinh}(k(z - h)). \hfill \\ \end{aligned}$$

7 $$\begin{aligned} b_{y} = \frac{{i\alpha_{S} Ae^{i(ky - \omega t)} }}{S \cdot \mathrm{sinh}(kh)}\left\{ {\zeta_{y} (z) \cdot F_{z} + i\psi_{y} (z) \cdot F_{y} } \right\}, \hfill \\ \zeta_{y} (z) = \mathrm{cosh}(\alpha_{S} z) + \frac{{\alpha_{S} }}{k}\mathrm{sinh}(\alpha_{S} z) - e^{kh} \left\{ {\mathrm{cosh}(\alpha_{S} (z - h)) - P \cdot \mathrm{sinh}(\alpha_{S} (z - h))} \right\} - \frac{kS}{{\alpha_{S} }}\mathrm{sinh}(k(z - h)), \hfill \\ \psi_{y} (z) = - P \cdot \left\{ {\mathrm{sinh}(\alpha_{S} z) + \frac{k}{{\alpha_{S} }}\mathrm{cosh}(\alpha_{S} z)} \right\} - e^{kh} \left\{ {\mathrm{cosh}(\alpha_{S} (z - h)) - P \cdot \mathrm{sinh}(\alpha_{S} (z - h))} \right\} + \frac{kS}{{\alpha_{S} }}\mathrm{cosh}(k(z - h)). \hfill \\ \end{aligned}$$

$${b}_{z}=\frac{kA{e}^{i(ky-\omega t)}}{S\cdot \mathrm{sinh}\left(kh\right)}\left\{{\zeta }_{z}\left(z\right)\cdot {F}_{z}+i{\psi }_{z}\left(z\right)\cdot {F}_{y}\right\},$$

$${\zeta }_{z}\left(z\right)=-\mathrm{sinh}\left({\alpha }_{s}z\right)-\frac{{\alpha }_{s}}{k}\mathrm{cosh}\left({\alpha }_{s}z\right)-{e}^{kh}\left\{P\cdot \mathrm{cosh}\left({\alpha }_{s}\left(z-h\right)\right)-\mathrm{sinh}\left({\alpha }_{s}\left(z-h\right)\right)\right\}+S\cdot \mathrm{cosh}\left(k\left(z-h\right)\right),$$

8 $${\psi }_{z}\left(z\right)=P\cdot \left\{\mathrm{cosh}\left({\alpha }_{s}z\right)+\frac{k}{{\alpha }_{s}}\mathrm{sinh}\left({\alpha }_{s}z\right)\right\}-{e}^{kh}\left\{P\cdot \mathrm{cosh}\left({\alpha }_{s}\left(z-h\right)\right)-\mathrm{sinh}\left({\alpha }_{s}\left(z-h\right)\right)\right\}-S\cdot \mathrm{sinh}\left(k\left(z-h\right)\right).$$

*α*_s_ is the EM wave number in the ocean and given by:

9 $$\alpha_{s} = \sqrt {(k^{2} - i\omega \mu_{0} \sigma_{s} )} .$$

The coefficients, *P* and *S*, convey information of the electrical substrata beneath the seafloor through a ratio, *R*:

10 $$R = :\frac{{D_{1} }}{{C_{1} }},P = \frac{{\alpha_{S} }}{{\alpha_{1} }}\frac{1 + R}{{1 - R}},S = \frac{{\alpha_{S} }}{k}\left( {1 + \frac{k}{{\alpha_{1} }}\frac{1 + R}{{1 - R}}} \right)\mathrm{cosh}(\alpha_{S} h) + \left( {1 + \frac{{\alpha_{S}^{2} }}{{k\alpha_{1} }}\frac{1 + R}{{1 - R}}} \right)\mathrm{sinh}(\alpha_{S} h).$$

*R* itself can be calculated by the following recursive relation with the EM wave number, *α*_*l*,_ in the *l*-th layer:

11 $$\left( {\begin{array}{*{20}c} {C_{l} } \\ {D_{l} } \\ \end{array} } \right) = \frac{1}{{2\alpha_{l} }}\left( {\begin{array}{*{20}c} {\alpha_{l} e^{{(\alpha_{l + 1} - \alpha_{l} )zl}} + \alpha_{l + 1} e^{{(\alpha_{l + 1} - \alpha_{l} )zl}} } & {\alpha_{l} e^{{ - (\alpha_{l + 1} + \alpha_{l} )zl}} - \alpha_{l + 1} e^{{ - (\alpha_{l + 1} + \alpha_{l} )zl}} } \\ {\alpha_{l} e^{{(\alpha_{l + 1} + \alpha_{l} )zl}} - \alpha_{l + 1} e^{{(\alpha_{l + 1} + \alpha_{l} )zl}} } & {\alpha_{l} e^{{ - (\alpha_{l + 1} - \alpha_{l} )zl}} + \alpha_{l + 1} e^{{ - (\alpha_{l + 1} - \alpha_{l} )zl}} } \\ \end{array} } \right)\left( {\begin{array}{*{20}c} {C_{l + 1} } \\ {D_{l + 1} } \\ \end{array} } \right)$$

The analytical solutions in eqs. (6) through (8) are a complete set of the two-dimensional wave field in the sense that the source electromotive force and the induced electric field lie in the *x*-direction alone to generate the magnetic field in the *yz*-plane. Each set of ζ_i_ and *ψ*_i_ (*i* = *x, y* or *z*) can be interpreted as transfer functions that represent the contribution of the ambient geomagnetic field (*F*_z_ or *F*_y_) to the tsunami-generated magnetic field, ***b***. The solutions are also ‘complete’ because they explicitly contain the contribution of *F*_y_ (i.e., *ψ*_i_), which has been neglected in most of the previous works. The contribution of the horizontal geomagnetic component in the background is especially important in the equatorial regions where the vertical geomagnetic component vanishes. We used eqs. (7) and (8) to make the plots in Fig. 2.

### Time domain modelling of tsunami-generated EM variations using solitary waves

The phase lead in the time domain of the magnetic variation can be investigated by semi-analytical modelling using solitary wave forms. Sea surface elevation of a solitary wave, $\eta$, can be expressed as

12 $$\eta (y,t)=A{^{\prime}}{\mathrm{sech}}^{2}\left(\frac{y-ct}{L}\right)$$

where $A{^{\prime}}$ is the peak height of the wave, $c$ is the phase velocity, $L$ is the horizontal scale length of the solitary wave. Defining the following Fourier transform and its inverse,

13 $$\stackrel{-}{f}\left(k,\omega \right)={\int }_{-\infty }^{\infty }dt{\int }_{-\infty }^{\infty }dy f\left(y,t\right)\mathrm{exp}\left(-iky+i\omega t\right)$$

14 $$f\left(y,t\right)={\left(2\pi \right)}^{-2}{\int }_{-\infty }^{\infty }d\omega {\int }_{-\infty }^{\infty }dk \stackrel{-}{f}\left(k,\omega \right)\mathrm{exp}\left(iky-i\omega t\right),$$

the solitary wave in the frequency-wave number domain is given by:

15 $$\stackrel{-}{\eta }\left(k,\omega \right)=\frac{2{\pi }^{2}A{L}^{2}k}{\mathrm{sinh}\left(\pi Lk/2\right)}\delta \left(\omega -kc\right).$$

The magnetic variation generated by the solitary wave can be evaluated by substitution of Eq. (15) into $A$ in Eq. (7) or (8) and their numerical integrations in Eq. (14). Figure 3 shows the results for ${b}_{z}$ by ${F}_{z}$ and ${F}_{y}$ and their phase relation in the time domain. In Fig. 3, superposition of two solitary waves is used for generation of the realistic tsunami first arrival, which includes a small initial negative polarity in the tsunami first arrival^24^. As a result, the adopted tsunami wave form is expressed by

16 $$\eta \left(y,t\right)=\sum_{i=1}^{2}{A}_{i}^{^{\prime}}{\mathrm{sech}}^{2}\left(\frac{y-ct-{y}_{i}}{{L}_{i}}\right),$$

where $({A{^{\prime}}}_{1},{A{^{\prime}}}_{2})=(1.1,-0.3)\mathrm{m}$, $\left({y}_{1}, {y}_{2}\right)=(1, 25)\mathrm{km}$, and ${L}_{1}={L}_{2}=18\mathrm{km}$. The resulting horizontal distance from the initial negative peak to the main positive one is 34 km. The characteristic frequency of the tsunami is therefore ~ 3 mHz, which is derived by Eq. (3) with the wavelength of 68 km.

## Supplementary information

Supplementary Information.
