# Supplementary material for: Tsunami-generated magnetic fields have primary and secondary arrivals like seismic waves
Source: Sci Rep. 2021 Jan 27;11:2287. doi: 10.1038/s41598-021-81820-5 (PMC7840733; doi:10.1038/s41598-021-81820-5)
Supplement: Supplementary file 1 — Supplementary Information. [file 41598_2021_81820_MOESM1_ESM.pdf]

Supplementary Information

for

"Tsunami-generated magnetic fields have primary and secondary arrivals like seismic waves"

by

Takuto Minami[1], Neesha R. Schnepf[2,3] & Hiroaki Toh[4,*]

[1]*Graduate School of Science, Kobe University, Nada-ku, Kobe 6578501, JAPAN*

[2]*Cooperative Institute for Research in Environmental Sciences (CIRES), University of Colorado, Boulder, CO 80309-0216 USA*

[3]*Dept. of Geological Sciences, University of Colorado, Boulder, CO 80309-0216 USA*

[4]*Graduate School of Science, Kyoto University, Sakyo-ku, Kyoto 6068502, JAPAN*

* *The corresponding author*

Results of tsunami kinetic simulation at the time of the 2011 Tohoku event

Kinetic simulation of the 2011 Tohoku tsunami event had already been done by one of the authors[3] using available data from DART stations as well as the linear Boussinesq approximation. The results are shown in Figs. S1a and b in the form of sea surface height. It is evident that the tsunamis propagated eastwards from the epicentre to the seafloor observation site, NWP, and thus the tsunami wavefront was almost completely oriented in the north-south direction around NWP.

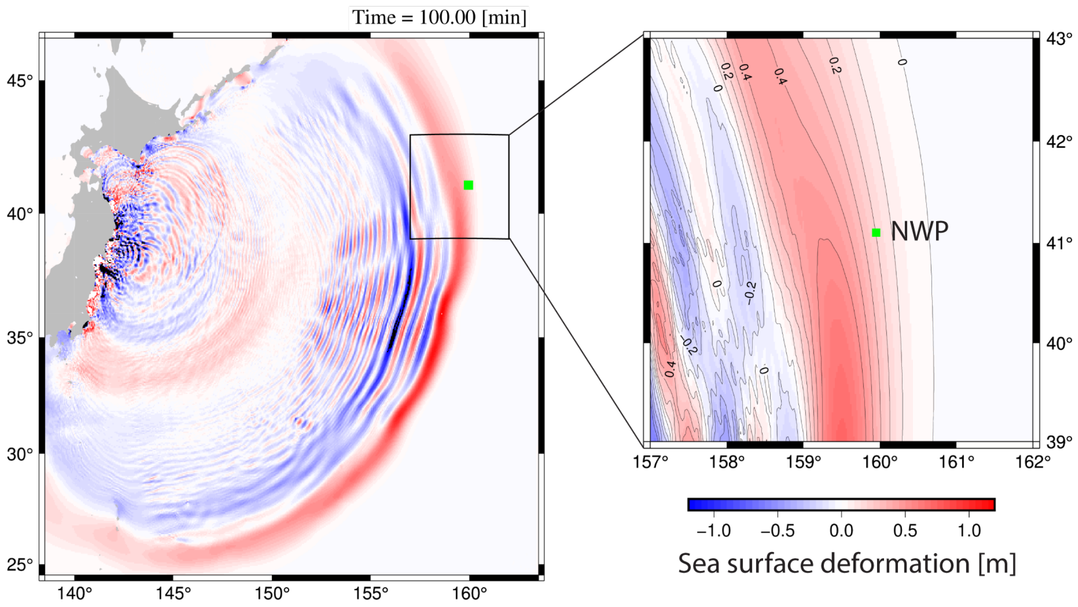

Fig. S1 **a,** A regional plot of the sea surface height [m] 100min after the earthquake origin time of 05:46UTC on March 11th, 2011 [Left]. **b,** A zoom-in plot of the sea surface height around NWP for the same instance as Fig. S1a [Right].

The maxima of v_y and v_z has a 90-degree phase difference

The phase difference between the v_zF_y coupling and the v_yF_z coupling is created by that between v_z and v_y. In the case of sinusoidal waves, the maximum speed of v_y collocates peaks of the wave height, while that of v_z is attained at nodes as shown in Fig. S2. This generates the 90-degree phase difference of b_z by v_z and by v_y described in the main text. In reality, b_z by v_y has a phase lead of 30-40 degrees against the maximum wave height as a result of coupling between the source electromotive force centered at the maximum wave height and the induced electric field in front of and behind the tsunami wave front in concern, while b_z by v_z has a 120-130° phase lead due to the 90-degree difference. However, if you compare their amplitudes, b_z by v_y is nearly 5 times as large as b_z by v_z as noted in the main text.

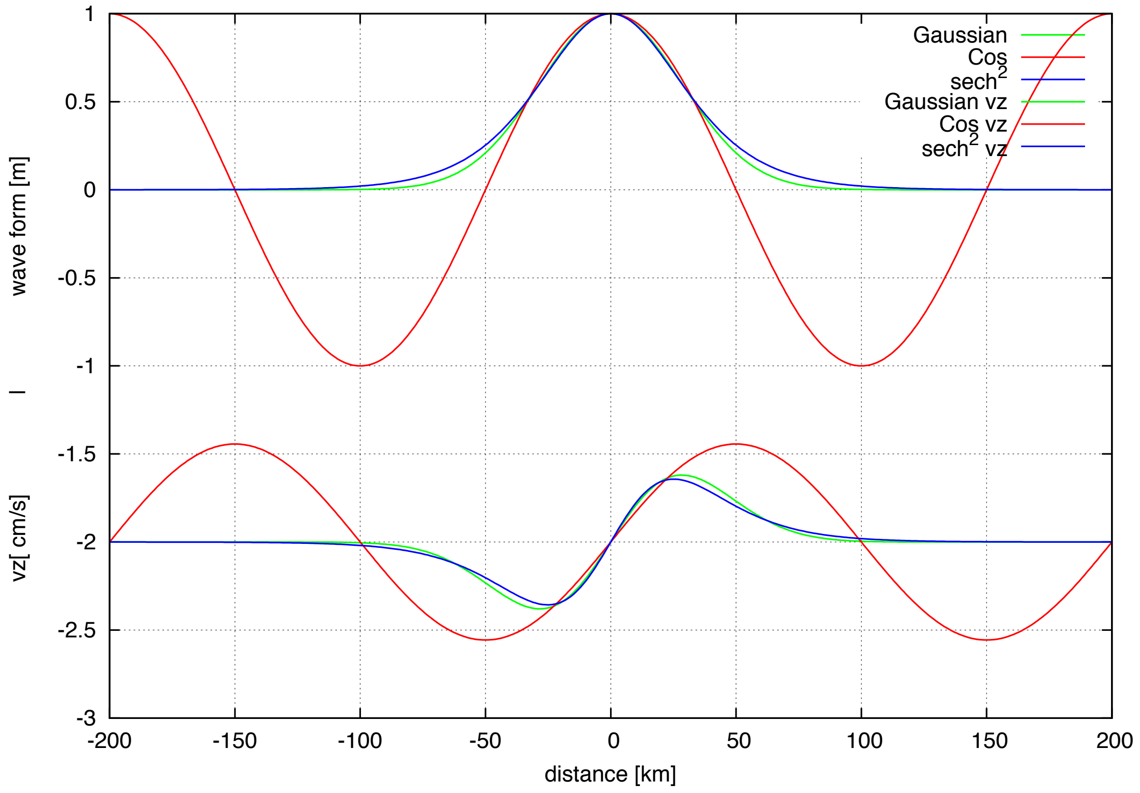

Fig. S2 [Upper] Wave forms of a sinusoidal wave (red) and two solitons (green or blue) with y [km] on the horizontal axis. A two-dimensional (2-D) Gaussian wave form and a solution of the 2-D KdV equation[ii] in the form of $sec^2(y)$ were chosen as solitons here. [Lower] v_z profiles of each wave form. Note that sinusoidal waves have the maximum v_z amplitude not at wave height peaks but at nodes, whereas that of v_y is attained at peaks. The phase difference, however, may become smaller in the case of solitons.

Amplitude ratio of the F_h to F_z on the Earth

In order to compare the v_zF_y contribution with the v_yF_z contribution quantitatively on the Earth, it is at least necessary to assess the ratio of the horizontal component (F_h) to the vertical component (F_z) of the present geomagnetic field.

Figure S3 shows the global distribution of the $| F_h / F_z |$ ratio calculated from a standard global geomagnetic field model[i]. It is evident that the ratio becomes larger than unity at low latitudes, which implies the importance of the v_zF_y coupling in those regions.

Note, however, that Fig. S3 does not show the $| F_y / F_z |$ ratio, because we do not know the tsunami propagation direction, viz., y-direction in the main text. It is also noteworthy that a east-west propagating tsunami may not have the v_zF_y contribution even on the geomagnetic equator, since the tsunami propagation is nearly pendicular to the geomagnetic lines of force in this special case.

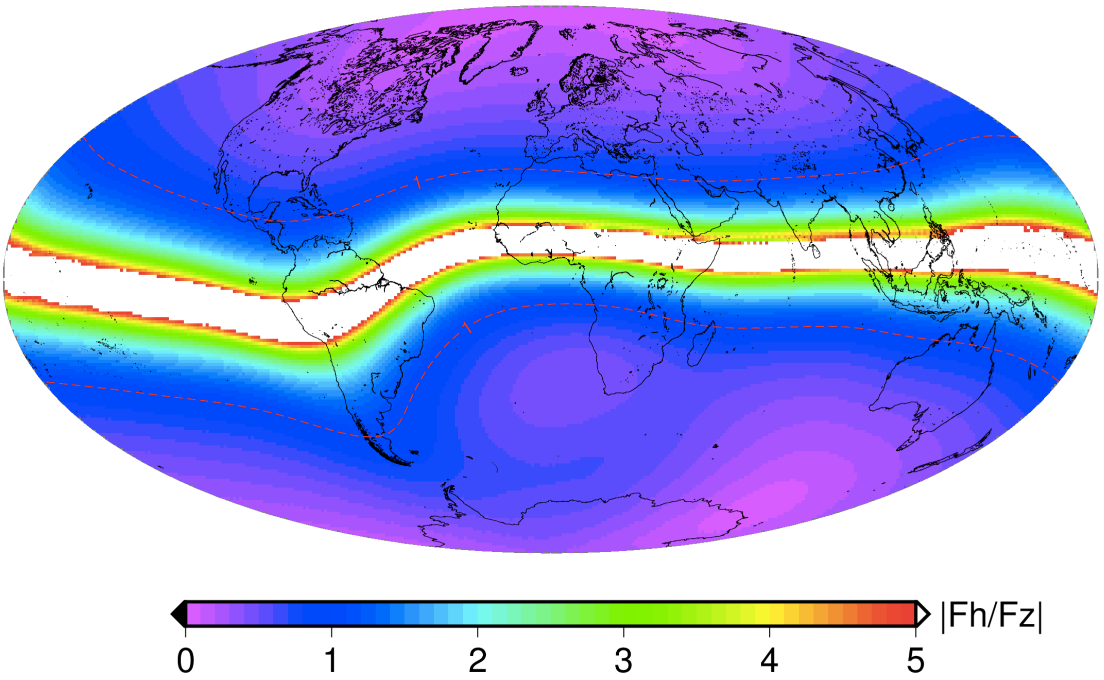

Fig. S3 Amplitude ratio of F_h to F_z at Epoch 2015.0. We used the 12th generation of International Geomagnetic Reference Field[i]. The red dashed lines are unity contours of the $| F_h / F_z |$ ratio, within which $| F_h |$ exceeds $| F_z |$. The white zone on both sides of the dip equator is a region of high $| F_h / F_z |$ ratios (>5).

Additional References

i. Thébault, E., et al. International Geomagnetic Reference Field: the 12th generation. Earth Planets Space (2015) 67:79. https://doi.org/10.1186/s40623-015-0228-9.

ii. Korteweg, D. J. & deVries, G. On the Change of Form of Long Waves Advancing in a Rectangular Canal, and on a New Type of Long Stationary Waves. *Phil. Mag. Series 5 (Taylor & Francis)* **39**, 422-443 (1895). doi:10.1080/14786449508620739

3. Refer to that in the main text.
